# Aggrecan and COMP Improve Periosteal Chondrogenesis by Delaying Chondrocyte Hypertrophic Maturation

**DOI:** 10.3389/fbioe.2020.01036

**Published:** 2020-08-28

**Authors:** Marjolein M. J. Caron, Maarten P. F. Janssen, Laura Peeters, Dominik R. Haudenschild, Andy Cremers, Don A. M. Surtel, Lodewijk W. van Rhijn, Pieter J. Emans, Tim J. M. Welting

**Affiliations:** ^1^Laboratory for Experimental Orthopedics, Department of Orthopedic Surgery, CAPHRI Care and Public Health Research Institute, Maastricht University Medical Center, Maastricht, Netherlands; ^2^Department of Orthopedic Surgery, University of California Davis School of Medicine, Sacramento, CA, United States

**Keywords:** chondrocyte hypertrophy, endochondral ossification, COMP, aggrecan, periosteum, periosteal chondrogenesis

## Abstract

The generation of cartilage from progenitor cells for the purpose of cartilage repair is often hampered by hypertrophic differentiation of the engineered cartilaginous tissue caused by endochondral ossification. Since a healthy cartilage matrix contains high amounts of Aggrecan and COMP, we hypothesized that their supplementation in the biogel used in the generation of subperiosteal cartilage mimics the composition of the cartilage extracellular matrix environment, with beneficial properties for the engineered cartilage. Supplementation of COMP or Aggrecan was studied *in vitro* during chondrogenic differentiation of rabbit periosteum cells and periosteum-derived chondrocytes. Low melting agarose was supplemented with bovine Aggrecan, human recombinant COMP or vehicle and was injected between the bone and periosteum at the upper medial side of the tibia of New Zealand white rabbits. Generated subperiosteal cartilage tissue was analyzed for weight, GAG and DNA content and ALP activity. Key markers of different phases of endochondral ossification were measured by RT-qPCR. For the *in vitro* experiments, no significant differences in chondrogenic marker expression were detected following COMP or Aggrecan supplementation, while *in vivo* favorable chondrogenic marker expression was detected. Gene expression levels of hypertrophic markers as well as ALP activity were significantly decreased in the Aggrecan and COMP supplemented conditions compared to controls. The wet weight and GAG content of the *in vivo* generated subperiosteal cartilage tissue was not significantly different between groups. Data demonstrate the potential of Aggrecan and COMP to favorably influence the subperiosteal microenvironment for the *in vivo* generation of cartilage for the optimization of cartilage regenerative approaches.

## Introduction

Cartilage lesions can be debilitating, and are a high-risk factor for the development of osteoarthritis (OA) over time (Mollenhauer and Erdmann, 2002). Cartilage lesions can be treated with surgical techniques such as microfracture (MF), mosaicplasty (MP), autologous chondrocyte implantation (ACI) or implantation of a small focal prosthesis (Hunziker, 2002). Donor site morbidity, limited donor cartilage availability, high costs, and inferior repair tissue quality, (respectively) are just some of the disadvantages of these approaches (Caldwell and Wang, 2014; Caron et al., 2014). We proposed a novel paradigm for *de novo* engineering of cartilaginous tissues, the *in vivo* bioreactor (IVB). This is an alternative cartilage repair concept that we aim to further develop (Emans et al., 2010). The IVB employs the fracture healing response as a way to generate autologous donor cartilage, suitable for implantation to repair (osteo)chondral defects (Emans et al., 2007, 2010). During bone fracture healing the local periosteum plays an important role in the healing process by providing periosteal mesenchymal progenitor cells, which differentiate into chondrocytes and form the cartilaginous callus tissue that remodels via endochondral ossification to ultimately heal the bone fracture (Nakahara et al., 1990; Jansen et al., 2008; Bahney et al., 2019). We discovered that local subperiosteal application of an agarose biogel provokes a similar cartilage callus-forming process within the created subperiosteal space, without the need of a fracture (Emans et al., 2010). This cartilaginous tissue presents all the hallmarks of hyaline cartilage, and upon transplantation, can heal an osteochondral defect out to 9 months in a rabbit model (Emans et al., 2010). However, without further optimization IVB-generated cartilage tissue is prone to further differentiate into hypertrophic cartilage, leading to unwanted ossification.

An important part of the dry-weight of articular cartilage consists of extracellular matrix (ECM) proteins (type II collagen (Col2a1), aggrecan (Acan), cartilage oligomeric matrix protein (COMP), etc.) (Mankin et al., 2000; Poole et al., 2001; Moreira-Teixeira et al., 2011). ECM proteins are thus important determinants in cartilage tissue homeostasis and their efficient synthesis is a prerequisite to creating cartilage volume. In addition, these major ECM protein species also condition the cartilage microenvironment in a unique way. Aggrecan plays a key role in generating the cartilage’s fixed negative charge due to its glycosaminoglycan content, leading to its water-attracting properties (Roughley and Mort, 2014), while COMP provides the cartilage with retention capacity for TGF-β superfamily member growth factors (Haudenschild et al., 2011). Therefore, we hypothesized that the supplementation of the IVB biogel with Aggrecan or COMP mimics the composition of the native cartilage extracellular matrix microenvironment, with the potential to gain control over the chondrogenic potential of the IVB.

## Materials and Methods

### Recombinant Expression and Purification of COMP

Full-length recombinant human (rh)COMP was prepared as previously described (Haudenschild et al., 2011). Briefly, human COMP cDNA was cloned into a pQE mammalian expression vector (Qiagen), which was then stably transduced into human HEK293T cells. Cells were expanded in in DMEM with 10% FBS until 15 cm tissue-culture dishes were 80% confluent, then the FBS was reduced to 0.1% FBS and conditioned media collected and replenished daily for up to 1 week. COMP was purified to near homogeneity from the conditioned culture media using nickel-nitrilotriacetic acid column affinity chromatography (Ni-NTA Agarose, Qiagen). The eluted protein was buffer-exchanged into 20 mM HEPES (pH 7.0), 2 mM CaCl_2_, and 500 mM NaCl, at approximately 500 μg/ml, with 30% glycerol added prior to storage at −80C.

### Periosteum Cell Culture

As previously described (Emans et al., 2006, 2010), the periosteum was harvested from the proximal tibia of New Zealand White Rabbits and cut into small pieces using a sterile surgical blade. Post-mortem animals were obtained from an unrelated study; no ethical approval was necessary. Periosteal pieces were digested for 3 h at 37°C in collagenase II solution [300 U/ml in HEPES buffered Dulbecco’s Modified Eagle Medium (DMEM; Invitrogen, Carlsbad, CA, United States) supplemented with 1% antibiotic/antimycotic (Invitrogen)] under continuous agitation. The preparation was rinsed with 0.9% NaCl over a 70 μm cell strainer and plated in culture flasks. Cells were cultured in a humidified atmosphere at 37°C, 5% CO_2_ in culture medium consisting of: Minimal Essential Medium (MEM)/D-valine (Invitrogen), 10% fetal calf serum (FCS) (Sigma-Aldrich, St Louis, MO, United States), 1% antibiotic/antimycotic (Invitrogen), 1% non-essential amino acids (NEAA, Invitrogen) and 2 mM l-glutamine (Sigma-Aldrich) (Gilbert and Migeon, 1975; Jansen et al., 2008). After reaching confluence, cells were passaged 1:2 until passage 2. Passage 2 rabbit periosteal cells from 1 donor were plated at 30.000 cells/cm^2^ in triplicates per condition and the next day chondrogenic differentiation was initiated by changing the culture medium to differentiation medium consisting of: Dulbecco’s Modified Eagle Medium (DMEM) high glucose (Invitrogen), 10% FCS (Sigma-Aldrich), 1% antibiotic/antimycotic (Invitrogen), 1 mM sodium pyruvate (Sigma-Aldrich), 1% insulin-transferrin-selenite solution (ITS; Sigma-Aldrich), 40 μg/ml L-proline (Sigma-Aldrich), 10 ng/ml TGF-β (Invitrogen), 25 μg/ml L-ascorbic acid-2-phosphate (Sigma-Aldrich), and 100 nM dexamethasone. Glycosaminoglycan containing bovine Aggrecan from articular cartilage (Sigma-Aldrich A1960) (Supplementary Figure S1) was added at 2 μg/ml concentration and rhCOMP was added at 200 μg/ml. The same volume of 0.9% sodium chloride was added as a control. Differentiation medium was changed every other day and after 0 (baseline measurement) and 21 days cells were harvested for RNA isolation and ALP activity.

### Chondrocytes Derived From IVB Cartilage

Cells were obtained from cartilage out of periosteum tissue generated *in vivo* in New Zealand White Rabbits (DEC2005-159) (Emans et al., 2007). The IVB cartilage tissue was harvested directly after euthanization. The autologous IVB cartilage was separated from the periosteum by dissecting with a scalpel and the overlying fibrous tissue was carefully removed. This cartilage tissue is distinct in phenotype and consistency so risk of contamination with other tissues in the sample is negligible. Tissue was digested for 3 h at 37°C in collagenase II solution [300 U/ml in HEPES buffered DMEM/F12 (Invitrogen) supplemented with 1% antibiotic/antimycotic (Invitrogen)] under continuous agitation. The preparation was rinsed with 0.9% NaCl over a 70 μm cell strainer and plated in culture flasks. Cells were cultured in a humidified atmosphere at 37°C, 5% CO_2_ in culture medium consisting of: DMEM/F12, 10% FCS, 1% antibiotic/antimycotic and 1% NEAA. After reaching confluence, cells were passaged 1:2 until passage 6. Passage 6 cells from 1 donor were plated at 30.000 cells/cm^2^ in triplicates per condition and the next day chondrogenic redifferentiation was initiated by changing the culture medium to redifferentiation medium consisting of: DMEM/F12, 1% antibiotic/antimycotic, 1% NEAA, 1% ITS, 10 ng/ml TGF-β and 25 μg/ml L-ascorbic acid-2-phosphate. Bovine Aggrecan was added at a 2 μg/ml concentration and rhCOMP was added at 200 μg/ml. The same volume of 0.9% sodium chloride was added as a control. Differentiation medium was changed every other day and after 0 (baseline measurement) and 7 days cells were harvested for RNA isolation.

### Animal Study

Twenty-four knees in 12 female, specific-pathogen-free (SPF) New Zealand White Rabbits were used for this experiment (Charles River Laboratories, Wilmington, MA, United States; 107 days old, ∼1.8 kg). The experiment was approved by the Maastricht University animal ethical committee (DEC 2012-151) and we confirm that all experiments were performed in accordance with relevant guidelines and regulations (ARRIVE). Throughout the experiment, animals were housed in groups under standard conditions with *ad libitum* access to water and food and 12 h of light each day. Animal well-being and behavior (score in response to stimuli, back arch, twitch, wincing, posture, self-care, condition of skin, mobility, limb loading, difficulties in respiration/breathing, dehydration or undernourishment symptoms, color of the mucous membranes and extremities, edema/swelling/cold feeling and other notable abnormalities) were checked daily. The sample size was calculated and corrected for potential dropout, and eight animals per group were included. The IVB method described by Emans and colleagues was used for ectopically inducing cartilage formation, in which a subperiosteal space is created to induce periosteal endochondral ossification (Emans et al., 2007, 2010; Janssen et al., 2017). In short, the skin was opened over the upper medial side of the tibia, the periosteum was incised just medially of the pes anserinus, leaving the semitendinosus tendon untouched. The periosteum was elevated proximally with a probe and 0.2 ml of a 2% (w/v) agarose-based gel (2 g of ultra-pure low-melting agarose granules (Cat no: 10975035, Lot No: MO91807; Invitrogen) in 100 ml of 0.9% NaCl, followed by steam-sterilization) was injected between the bone and periosteum. Bovine Aggrecan was added at a 2% w/v or rhCOMP was added at 0.5 mg/ml to the agarose-gel. The wound was closed in separate layers with Vicryl Rapide™ 4–0 absorbable sutures (Ethicon, Kirkton, United Kingdom). This procedure was repeated on the contralateral tibia. After 14 days, rabbits were euthanized by an overdose of intravenous pentobarbital. The IVB cartilage tissue was harvested directly after euthanization. The autologous IVB cartilage was separated from the periosteum by dissecting with a scalpel and the overlying fibrous tissue was carefully removed. This cartilage tissue is distinct in phenotype and consistency so risk of contamination with other tissues in the sample is negligible. Generated subperiosteal cartilage tissue was analyzed for weight, glycosaminoglycan (GAG)- and DNA content. In addition, samples were taken for gene expression analysis and ALP activity assay.

### Gene Expression Analysis

Cells and ectopically formed cartilage tissue on the tibia were harvested and lysed in TRIzol (Life Technologies| Thermo Fisher Scientific, Carlsbad, CA, United States). RNA isolation, RNA quantification by ultraviolet (UV) spectrometry (Biodrop; Isogen Life Sciences, Utrecht, Netherlands) and cDNA synthesis were performed as described before (Welting et al., 2011; Caron et al., 2012). Real-time quantitative PCR (RT-qPCR) was performed using Takyon No ROX Sybr^®^ Green MasterMix blue dTTP (Eurogentec, Seraing, Belgium). A CFX96 RealTime PCR Detection system (Biorad, Hercules, CA, United States) was used for amplification with the following protocol: initial denaturation 95°C for 10 min, followed by 40 cycles of amplification (denaturation 15 s at 95°C and annealing 1 min at 60°C). Validated primer sequences used are listed in Table 1. Data were analyzed using the standard curve method, mRNA expression was normalized to the reference gene (28S rRNA) and gene expression was calculated as fold change as compared to baseline conditions (*in vitro* studies) or control conditions (*in vivo* study).

**TABLE 1:** Primer sequences for RT-qPCR.

Oligo sets	Forward	Reverse	−ΔCt in differentiated periosteal cells	−ΔCt in IVB generated chondrocytes	−ΔCt in IVB generated cartilage
Acan	CGGGACACCAACGAGACCTAT	CTGGCGACGTTGCGTAAAA	−11,48	−14,88	−16,76
Alpl	GGAGGATGTGGCCGTCTTC	CTGCGTAAGCCATCACATGAG	−14,55	−12,82	−14,09
Nkx3-2	ACCTGGCAGCTTCGCTGAA	AGGTCGGCGGCCATCT	−21,15	−19,85	−25,96
BMP2	AGAAAAGCGTCAAGCGAAACA	GTCCACGTACAAAGGGTGTCTCT	−13,04	−12,67	−19,46
Col1a1	CTGACTGGAAGAGCGGAGAGTAC	CCATGTCGCAGAAGACCTTGA	−14,19	−14,39	−13,85
Col2a1	TGGGTGTTCTATTTATTTATTGTCTTCCT	GCGTTGGACTCACACCAGTTAGT	−11,28	−10,77	−16,57
Col10a1	AACCTGGACAACAGGGACTTACA	CCATATCCTGTTTCCCCTTTCTG	−13,50	−10,59	−18,82
Cox-2	ACCAACATGATGTTTGCATTCTTT	GGTCCCCGCTTAAGATCTGTCT	−17,27	−13,32	−21,20
ID2	CCCGATGAGCCTGCTATACAA	TGGGCACCAGCTCCTTGA	−14,85	−16,21	−17,42
Mmp13	CGATGAAGACCCCAACCCTAA	ACTGGTAATGGCATCAAGGGATA	−13,65	−17,13	−18,45
PTHrP	AAGGGCAAGTCCATCCAAGA	CTCGGCGGTGTGTGGATTTC	−12,53	−14,11	−22,40
Runx2	TGATGACACTGCCACCTCTGA	GCACCTGCCTGGCTCTTCT	−18,25	−13,94	−18,23
Smad7	GCAACCCCCATCACCTTAGTC	GTTTGAGAAAATCCATTGGGTATCTG	−15,33	−13,66	−15,38
Sox9	AGTACCCGCACCTGCACAAC	CGCTTCTCGCTCTCGTTCAG	−15,03	−12,24	−17,86
TGFb3	ACTTGCACCACCTTGGACTTC	GGTCATCACCGTTGGCTCA	−15,85	−11,76	−16,00
28S rRNA	GCCATGGTAATCCTGCTCAGTAC	GCTCCTCAGCCAAGCACATAC	Reference	Reference	Reference

*The 5′ to 3′ forward and reverse oligonucleotide sequences used for RT-qPCR are listed in the table. The −ΔCt values for the control condition in the *in vivo* IVB generated cartilage tissue, periosteal cells and IVB-derived chondrocytes are shown.*

### sGAG Assay

The total sulfated glycosaminoglycan (sGAG) content of the ectopically formed cartilage tissue was measured using a standardized modified 1,9-dimethyl methylene blue (DMMB) assay (Polysciences) (Farndale et al., 1982, 1986). The absorbance of samples was read at 540 and 595 nm using a spectrophotometer (Multiskan FC, ThermoFisher Scientific). GAG concentrations were calculated using a standard curve of chondroitin sulfate (Sigma-Aldrich). GAG content was normalized for total DNA content or wet weight of the ectopically formed cartilage tissue.

### DNA Quantification

The DNA concentration was determined using SYBR^®^ Green I Nucleic Acid stain (Invitrogen). A serially diluted standard curve of genomic control DNA (calf thymus, Invitrogen) in TE buffer (10 mM Tris/HCl pH 8.0, 1 mM EDTA) was included to quantify the DNA concentration in the samples. Before measurement, samples were diluted in TE buffer (1 μl sample and 99 μl TE buffer) and standards were prepared. SYBR^®^ Green was diluted 10,000 times in TE buffer and 100 μl of this solution was added to 100 μl of the above-prepared samples or standards. Fluorescence was determined in standard 96-well ELISA plates in a Spectramax M2 microplate reader (Molecular Devices, Sunnyvale, CA, United States): excitation 488 nm and emission 522 nm.

### ALP Activity Assay

Cells or cartilage tissues were lysed in 1.5 M Tris–HCl pH 9.0; 2% (v/v) Triton X-100 and homogenized by sonication (Soniprep 150 MSE). Insoluble material was removed by centrifugation (5 min; 13,000 × *g*; 4°C). Total protein concentration was determined BCA assay (Sigma-Aldrich). ALP enzyme activity in-time was measured by ALP-depend enzymatic conversion of p-nitrophenyl phosphate to p-nitrophenol in buffer containing 1.5 M Tris–HCl; pH 9.0, 1 mM ZnCl_2_, 1 mM MgCl_2_, and 7.5 mM p-nitrophenyl phosphate. Substrate conversion was spectrophotometrically quantified at 405 nm and p-nitrophenol concentrations were determined via a p-nitrophenol calibration series. Values were normalized to total protein concentration and ALP enzyme activity was calculated as mmol/min/μg.

### Statistics

**FIGURE 1:**
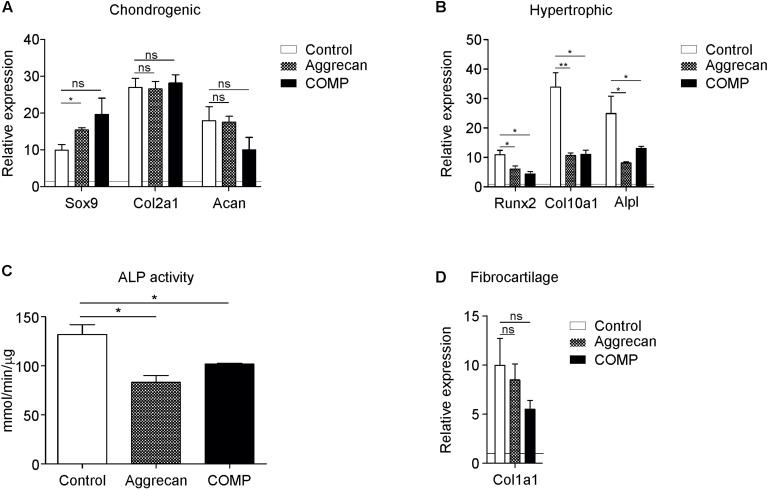
Addition of Aggrecan or COMP during chondrogenic differentiation of rabbit periosteal cells results in decreased hypertrophic differentiation. Periosteal derived cells differentiated in chondrogenic lineage under control conditions (white bars) and with Aggrecan (2 μg/ml; dotted bars) or COMP (200 μg/ml; black bars) for 21 days. **(A)** Induction of chondrogenic markers Sox9, Col2a1, and Acan mRNA expression was determined by RT-qPCR, normalized for 28S rRNA expression and set relative to baseline (*t* = 0) values (indicated by horizontal line). **(B)** Induction of hypertrophic markers Runx2, Col10a1, and Alpl mRNA expression was determined similarly to samples from **(A)**. **(C)** ALP enzyme activity in cell lysates of same conditions was determined and normalized to total protein content. **(D)** Fibrocartilage marker Col1a1 mRNA expression as determined in similarly to from **(A)**. In graphs, error bars represent mean ± SEM. Statistically significant differences (*p* < 0.05) are shown by an *, ** = *p* < 0.01, ns = not significant.

**FIGURE 2:**
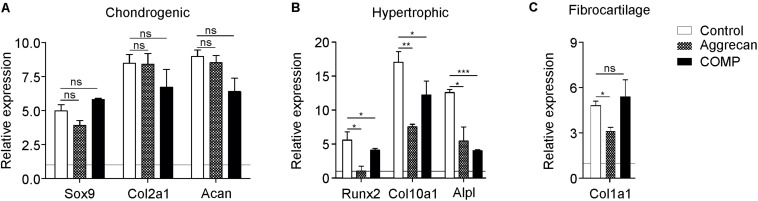
Better cartilage quality of chondrocytes generated from periosteal tissue when exposed to Aggrecan or COMP. Chondrocytic cells derived from ectopic generated cartilage out of periosteum tissue were redifferentiated under control conditions (white bars) and with Aggrecan (2 μg/ml; dotted bars) or COMP (200 μg/ml; black bars) for 7 days. **(A)** Induction of chondrogenic markers Sox9, Col2a1, and Acan mRNA expression was determined by RT-qPCR, normalized for 28S rRNA expression and set relative to baseline (*t* = 0) values (indicated by horizontal line). **(B)** Induction of hypertrophic markers Runx2, Col10a1, and Alpl mRNA expression was determined similarly to samples from **(A). (C)** Fibrocartilage marker Col1a1 mRNA expression was determined similarly to samples from **(A)**. In graphs, error bars represent mean ± SEM. Statistically significant differences (*p* < 0.05) are shown by an *, ** = *p* < 0.01, *** = *p* < 0.0001, ns = not significant.

**FIGURE 3:**
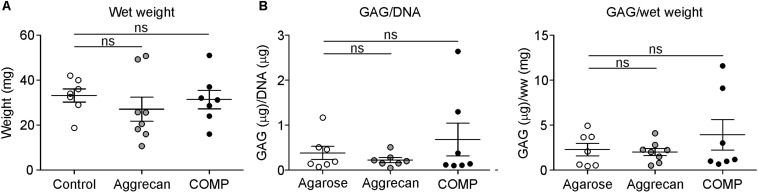
Similar GAG content in cartilage generated out of periosteum *in vivo*. Cartilage formation was ectopically induced by injecting an agarose biogel (*n* = 7) with or without the addition of Aggrecan (2% w/v; *n* = 8) or COMP (0.5 mg/ml; *n* = 7) under the tibial periosteum of rabbits and after 14 days generated tissue was harvested for analysis. **(A)** Wet weight was determined for each ectopically generated cartilaginous tissue. **(B)** GAG content corrected for DNA content (left panel) or for wet weight (right panel) was determined in samples from **(A)**. Each dot represents the determined value for each of these individual generated tissues per group and lines in graphs indicate mean ± SEM. Statistically significant differences (*p* < 0.05) are shown by an *, ns = not significant.

**FIGURE 4:**
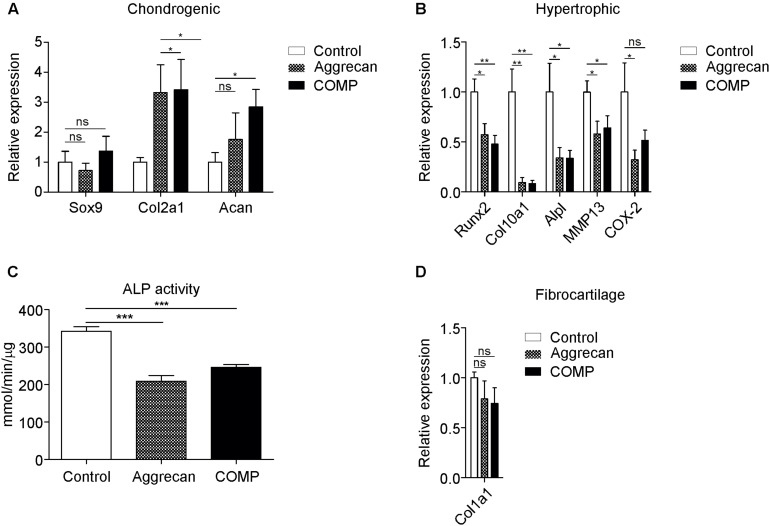
Decreased hypertrophic marker expression in *in vivo* generated cartilage stimulated with Aggrecan or COMP. Cartilage formation was ectopically induced by injecting an agarose biogel (*n* = 8) with or without the addition Aggrecan (2% w/v; *n* = 8) or COMP (0.5 mg/ml; *n* = 8) under the tibial periosteum of rabbits and after 14 days generated tissues were harvested for gene expression analysis. **(A)** Induction of chondrogenic markers Sox9, Col2a1, and Acan mRNA expression was determined by RT-qPCR and normalized for 28S rRNA expression. **(B)** Induction of hypertrophic markers Runx2, Col10a1, and Alpl mRNA expression was determined by RT-qPCR at day 14 and normalized for 28S rRNA expression. **(C)** ALP enzyme activity in tissue lysates of same conditions was determined and normalized to total protein content. **(D)** Fibrocartilage marker Col1a1 mRNA expression as determined by RT-qPCR and normalized to 28S rRNA expression. In graphs, error bars represent mean ± SEM. Statistically significant differences (*p* < 0.05) are shown by an *, ** = *p* < 0.01, *** = *p* < 0.005, ns = not significant.

**FIGURE 5:**
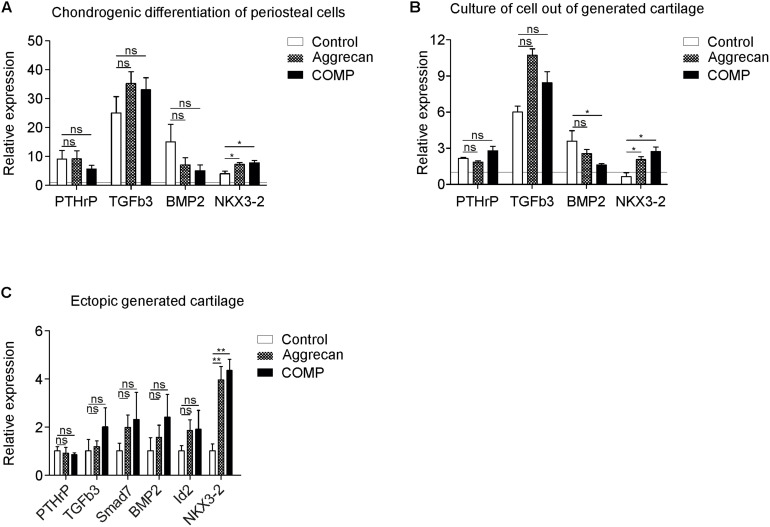
Increased NKX3-2 mRNA expression in COMP and Aggrecan generated cartilage *in vitro* and *in vivo*. **(A)** Expression of PTHrP, TGFb3, BMP2, and NKX3-2 was determined by RT-qPCR, normalized for 28S rRNA expression and set relative to baseline (*t* = 0) values (indicated by horizontal line) in samples from Figure 1 (chondrogenic differentiation of periosteal cells). **(B)** Expression of PTHrP, TGFb3, BMP2, and NKX3-2 was determined by RT-qPCR, normalized for 28S rRNA expression and set relative to baseline (*t* = 0) values (indicated by horizontal line) in samples from Figure 2 (redifferentiation of cells isolated from ectopically generated cartilage). **(C)** Expression of PTHrP, TGFb3, Smad7, BMP2, Id2, NKX3-2 was determined by RT-qPCR and normalized for 28S rRNA expression in samples from Figure 4 (ectopically generated cartilage *in vivo*). White bars represent the control condition, dotted bars the condition supplemented with Aggrecan and the black bars the condition supplemented with COMP. In graphs, error bars represent mean ± SEM. Statistically significant differences (*p* < 0.05) are shown by an *, ** = *p* < 0.01, ns = not significant.

Statistical significance (*p* < 0.05) was determined by student’s two-tailed *t*-test for *in vitro* experiments shown in Figures 1, 2, and 5 using Graphpad PRISM 5.0 (La Jolla, CA, United States). Due to limited sample size (triplicates), normal distribution of input data was assumed as normality could not be reliably tested. For the *in vivo* experiment, normal distribution of input data was tested by D’Agostino-Pearson omnibus normality tests and all data from the *in vivo* study (Figures 3–5) passed the normality tests. Statistical significance (*p* < 0.05) was determined by student’s two-tailed *t*-test. Lines in graphs represent mean ± standard error of the mean (SEM).

## Results

### Addition of Aggrecan or COMP During Chondrogenic Differentiation of Periosteal Progenitor Cells Inhibits Chondrocyte Hypertrophy

As the IVB relies on chondrogenic differentiation of the local periosteum, we determined if COMP or Aggrecan could improve the chondrogenic differentiation of periosteum-derived progenitor cells *in vitro*. These two cartilage ECM components were added to the chondrogenic differentiation media of rabbit periosteal derived cells. After 21 days, differences in chondrogenic and hypertrophic gene expression were analyzed between groups. Expression of SRY (sex-determining region Y) box9 (Sox9) was significantly increased by Aggrecan (Figure 1A), but not significantly by COMP. No significant differences were found in the expression of Col2a1 and Aggrecan by supplementation of either Aggrecan or COMP (Figure 1A). In contrast, gene expression of hypertrophic markers was all significantly repressed by Aggrecan or COMP exposure during chondrogenic differentiation of periosteum cells (Figure 1B). Runt-related transcription factor 2 (Runx2) expression was reduced in Aggrecan and COMP conditions. Collagen type X (Col10a1) and alkaline phosphatase (Alpl) expression responded similar as Runx2, with decreased gene expression after 21 days of exposure to Aggrecan or COMP (Figure 1B). This inhibition of hypertrophic maturation in chondrogenic differentiation of periosteal cells by Aggrecan or COMP was further confirmed by a significant decrease in ALP enzyme activity (Figure 1C). No significant differences were detected for fibrotic marker collagen type I (Col1a1) expression between groups (Figure 1D). Collectively, these data indicate that exposure of chondrogenically differentiating periosteum cells to supplemented Aggrecan or COMP does not influence the expression of key chondrogenic markers, but specifically suppresses chondrocyte hypertrophic differentiation in these *in vitro* cell cultures.

### Improved Chondrocyte Phenotype of Chondrocytes Derived From IVB Cartilage When Exposed to Aggrecan or COMP

The biogel (and additives in it) used for the IVB technique is expected to not only influence the initiation of chondrogenic differentiation but also aiding in maintaining or supporting the chondrogenic differentiation status of mature chondrocytes. Therefore, we likewise determined the effect of Aggrecan or COMP on chondrocytes that were isolated from IVB-generated cartilage from a previous *in vivo* experiment (Emans et al., 2007). After 7 days of culture with either Aggrecan or COMP, the chondrocyte phenotype was assessed by gene expression analysis. No major differences were observed in mRNA expression of chondrogenic markers Sox9, Col2a1, and Acan following the addition of Aggrecan or COMP to these cultures (Figure 2A). However, and in concert with results found above (Figure 1), the addition of Aggrecan or COMP to these cultures had a profound consequence for chondrocyte hypertrophy (Figure 2B). Expression of Runx2 was significantly decreased by Aggrecan or by COMP at day 7 in culture (Figure 2B). Significant repression of Col10A1 and Alpl was also observed following Aggrecan or COMP supplementation (Figure 2B). Col1a1 expression was significantly inhibited by Aggrecan in these cultures, however, not by COMP (Figure 2C). Together, these data indicate that Aggrecan and COMP improve the chondrocyte phenotype *in vitro* of mature chondrocytes isolated from IVB-generated cartilage by selectively decreasing chondrocyte hypertrophy.

### Quality of IVB Cartilage Generated With Aggrecan or COMP Supplementation of the Biogel

We next determined if Aggrecan or COMP supplementation to the IVB biogel leads is beneficial for the quality of ectopically generated cartilage in the IVB. We used the IVB technique as described earlier (Emans et al., 2010; Janssen et al., 2017) and added either Aggrecan (2% w/v; *n* = 8 IVBs) or COMP (0.5 mg/ml; *n* = 8 IVBs) to the agarose biogel, and compared the quality of the cartilage that was generated 14 days after creation of the IVBs with a control group in which only the empty agarose biogel condition was tested (*n* = 8 IVBs). The wet weight of the formed IVB tissues was not significantly different between the empty agarose group versus the IVBs in which the biogel was supplemented with Aggrecan or COMP (Figure 3A). Also, no significant differences between the control group and Aggrecan or COMP groups were found in the DNA content of the IVB generated tissues (data not shown). When GAG content in the IVB generated tissues was determined and normalized for either DNA content or tissue wet weight, again no significant differences were found between the groups (Figure 3B).

To analyze the IVB-generated ectopic cartilage tissues in more bio-molecular detail we determined the expression of chondrogenic and chondrocyte hypertrophy genes. Sox9 expression in the generated cartilage tissues was not significantly different between the control and Aggrecan-supplemented or between control and COMP-supplemented groups (Figure 4A). Expression of Col2a1 and Acan was significantly increased in the IVBs supplemented with COMP. The IVBs supplemented with Aggrecan showed a significantly increased Col2a1 expression. However, the increase in Acan expression was not significant (Figure 4A). In full agreement with data obtained from above *in vitro* cultures of periosteal chondrogenesis (Figure 1) and the IVB-derived chondrocytes (Figure 2), the most profound differences in gene expression were found for chondrocyte hypertrophy genes (Figure 4B). Runx2, Col10a1, and Alpl expression were significantly suppressed in the IVBs supplemented with Aggrecan or COMP (Figure 4B). When analyzing other chondrocyte hypertrophy-associated genes such as matrix metalloproteinase 13 (MMP13) or cyclooxygenase 2 (COX-2) (Welting et al., 2011), we observed that MMP13 expression was inhibited in both the Aggrecan and COMP groups, while COX-2 expression was reduced, but not significantly (Figure 4B). This inhibition of hypertrophic maturation of the IVB-generated ectopic cartilage by Aggrecan or COMP was further confirmed by a significant decrease in ALP enzyme activity (Figure 4C). No significant differences were found for Col1a1 expression between groups (Figure 4D). Overall, these results demonstrate that the supplementation of Aggrecan or COMP to the IVB agarose biogel does not change the quantity or GAG content of the generated cartilaginous tissues. However, gene expression analysis shows the development of a favorable cartilage phenotype, with a specific reduction of the magnitude of chondrocyte hypertrophy in the Aggrecan and COMP groups.

### Increased NKX3-2 mRNA Expression Following Aggrecan or COMP Supplementation

We next elucidated a potential biomolecular mechanism explaining the observed change in chondrogenic outcome in the chondrogenically differentiating periosteal cells, IVB-derived chondrocytes, and in the newly generated IVB tissues, as a result of exposure to Aggrecan or COMP. To this end, gene expression of important paracrine regulators (PTHrP, TGF-β3, and BMP2) of chondrogenic differentiation was determined (Kronenberg, 2003; Ripmeester et al., 2018). In addition, mRNA expression levels of Bagpipe Homeobox Protein Homolog 1 (Bapx1)/Homeobox Protein NK-3 Homolog B (NKX3-2), a transcriptional repressor of chondrocyte hypertrophic differentiation (Provot et al., 2006; Caron et al., 2013a), was determined in these samples.

At day 21 in *in vitro* chondrogenic differentiation of periosteal cells, expression of parathyroid hormone-related peptide (PTHrP), TGF-β3 and bone morphogenetic protein 2 (BMP2) was not significantly different between groups (Figure 5A). However, expression of NKX3-2 mRNA was significantly increased in the chondrogenic cultures supplemented with Aggrecan or COMP (*p* = 0.0300) (Figure 5A). Mature chondrocytes that were isolated from IVB cartilage and cultured *in vitro* for 7 days in the presence of Aggrecan or COMP did not show any significant responses of PTHrP or TGF-β3 (Figure 5B). Exposure of these cultures to COMP resulted in significant inhibition of BMP2 expression, while supplementation of Aggrecan to these cultures did not significantly alter BMP2 expression (Figure 5B). However, and similar to chondrogenesis of periosteal cells above, the gene expression of NKX3-2 was significantly increased in cultures supplemented with Aggrecan or COMP (Figure 5B).

In the *in vivo* ectopically generated IVB cartilage tissues in which the biogel was supplemented with Aggrecan or COMP, expression of PTHrP was not significantly different when compared to the control empty agarose biogel group (Figure 5C). Expression of TGFβ3 and TGFβ target gene Smad7 was not significantly altered in IVBs supplemented with Aggrecan or COMP (Figure 5C). BMP signaling, measured by BMP2 and DNA-binding protein inhibitor 2 (Id2) gene expression was not significantly different between groups (Figure 5C). Similar to above NKX3-2 expression data and its chondrocyte hypertrophy-suppressive action, NKX3-2 mRNA expression was significantly increased in the IVB cartilage tissues generated from biogel supplemented with Aggrecan or COMP. In conclusion, the gene expression of the chondrocyte hypertrophy transcriptional repressor NKX3-2 was significantly increased in all conditions supplemented with Aggrecan or COMP.

## Discussion

The goal of ectopic cartilage regeneration is to create sufficient quantity of hyaline cartilage of good quality to be used for transplantation. Limitations in quantity and progression into hypertrophy remain important drawbacks that need to be addressed in the field. In this study, we showed in three independent models that chondrogenic differentiation and cartilage homeostasis of periosteal cells *in vitro* and *in vivo* can be sustained by the supplementation of Aggrecan or COMP. It specifically leads to suppression of hypertrophic differentiation of the cartilaginous tissue, with possible involvement of NKX3-2 (Caron et al., 2013a, 2015).

Aggrecan is a key GAG-containing proteoglycan in cartilage and plays an important role in stabilizing the ECM in articular cartilage. Furthermore, due to negatively charged anionic groups of its GAG sidechains, aggrecan creates a large osmotic gradient which draws water into the tissue. This gives cartilage its unique properties (Kiani et al., 2002). Several studies have shown that articular chondrocytes and chondrogenically differentiating progenitor cells are osmolarity-responsive and increase their ECM synthesis under chondrocyte-physiological osmolarity (Urban et al., 1993; Palmer et al., 2001; Caron et al., 2013b), or after addition of oversulphated polysaccharides (Merceron et al., 2012). Likewise, plating of fibroblasts on an Aggrecan-coated surface (in the presence of TGF-β) was able to induce chondrogenic differentiation (French et al., 2004) of these cells. We hypothesized that the addition of Aggrecan to the *in vitro* cultures of differentiating periosteal cells, IVB-derived chondrocytes and eventually also the IVB-generated cartilage tissue, would increase the chondrogenic differentiation capacity of these cells. In the *in vitro* cultures we did not observe significantly increased chondrogenic marker expression as measured by Sox9, Col2a1, and Acan, However, in the IVB-generated cartilaginous tissue, the gene expression of Col2a1 was significantly increased by Aggrecan supplementation. GAG-bound TGF-β is able to stimulate neocartilage formation (Park et al., 2008) and it was recently shown that under cartilage physiological osmolarity TGF-β signaling was increased. This was associated with an improved chondrocyte phenotype (Tan Timur et al., 2019). Indeed, also in our studies we observed increased (but not significant) TGF-β3 expression in the Aggrecan-supplemented conditions. However, we were not able to determine if the actual osmolarity of the culture conditions was significantly increased due to Aggrecan supplementation. Interestingly, in the Aggrecan-supplemented conditions we observed a significant repression of chondrocyte hypertrophy (Runx2, Col10a1, Alpl expression, and ALP enzyme activity) in all three models. These data demonstrate that periosteal chondrogenic differentiation *in vitro* and *in vivo* and homeostasis of IVB-derived chondrocytes can be influenced in a hypertrophy-suppressive manner by supplementation with Aggrecan. NKX3-2 is known as a key transcriptional repressor of Runx2 during both early and late chondrogenic differentiation (Provot et al., 2006; Rainbow et al., 2014), providing control over hypertrophic differentiation. NKX3-2 mRNA expression was significantly upregulated in the aggrecan-supplemented cultures. To the best of our knowledge, it is unknown how Aggrecan would be able to induce the expression of NKX3-2 mRNA in these cells. GAGs are described to be able to bind and regulate activity of growth factors, chemokines, cytokines and adhesion molecules (Hadler-Olsen et al., 2011). For instance, FGF and VEGF are stored, stabilized and protected from degradation in the ECM trough interactions with GAGs, and upon stimulation can be released to exert their function (Jackson et al., 1991; Vlodavsky et al., 2006). We speculate that certain NKX3-2-inducing morphogens, such as Shh, PTHrP or BMPs (Zeng et al., 2002; Provot et al., 2006; Caron et al., 2015) are being retained by the GAG-containing supplemented Aggrecan (Forsten-Williams et al., 2008), potentially potentiating their activity and leading to a hypertrophy-suppressing action via NKX3-2. Indeed, our supporting data from ATDC5 chondrogenic differentiation suggest a role for NKX3-2 in hypertrophic differentiation via specific morphogens and increased osmolarity (Supplementary Figure S2). However, NKX3-2 data in our present study are limited by a current lack of evidence on the protein level in periosteal cells and needs further investigation to corroborate this hypothesis.

COMP is one of the thrombospondin proteins (TSP-5) that acts as a key component in the synthesis and homeostasis of the cartilage ECM (Acharya et al., 2014). COMP is essential in chondrogenic growth plate development (DiCesare et al., 1995; Rock et al., 2010) and mutations in COMP are linked to the human skeletal disorders pseudoachondroplasia (PSACH) and multiple epiphyseal dysplasia (MED) (Briggs et al., 1995; Hecht et al., 1995). In addition, elevation of COMP levels increased chondrogenic differentiation of human bone marrow stem cells (Acharya et al., 2014). In this study, however, supplementation with COMP did not lead to significant differences in Col2a1 and Acan expression in chondrogenically differentiating periosteal cells or cultures of chondrocytes derived from IVB cartilage tissue *in vitro*. In our *in vivo* study, however, IVB biogel supplementation with COMP did significantly increase the expression of chondrogenic markers Col2a1 and Acan. In analogy with the Aggrecan supplemented condition above, supplementation with COMP significant suppressed chondrocyte hypertrophy in all three tested chondrocyte models. COMP is a homopentamer acting as a key intermolecular bridge in cartilaginous tissues (Acharya et al., 2014). COMP is described to interact with cartilage ECM proteins, including collagen type 2 and Aggrecan, and as such plays a role in matrix assembly and tissue homeostasis. COMP also interacts with endogenous growth factors, such as TGFβs and BMPs, and acts as a lattice for their presentation to cells (Haudenschild et al., 2011; Ishida et al., 2013; Acharya et al., 2014). This influences, for instance, growth factor signaling and cell differentiation processes. The activity of TGFβ1 is potentiated when bound to COMP (Haudenschild et al., 2011), potentially explaining its prochondrogenic and hypertrophy-suppressing properties in our IVB experiments. It can also be noted that COMP binds BMP7 (Haudenschild et al., 2011). Previously we reported that BMP7 suppresses chondrocyte hypertrophy in an NKX3.2 dependent fashion (Caron et al., 2013a, 2015), and we consider a BMP7 activity-potentiating role for COMP as a possible explanation for our observations. Data supporting a role for TGFβ and BMP7 in the induction of NKX3-2 levels during ATDC5 chondrogenic differentiation are presented in Supplementary Figure S2.

Due to a limited quantity of IVB-generated cartilage tissue in this study, we needed to select the most insightful manner of analysis. Although posing a study limitation from a histological perspective, we preferred a quantitative analysis over histology and used gene expression, GAG content, DNA content, ALP activity, and wet weight as primary read-out parameters. Also, we could only test periosteal progenitor cells and chondrocytes from periosteal cartilage from one donor each. Despite these limitations, this study demonstrates in different models that conditioning of the micro-environment with cartilage ECM components Aggrecan or COMP creates a hypertrophy-suppressive niche with prochondrogenic properties for development of cartilaginous tissue in the IVB. A more prolonged analysis of the stability of the IVB neocartilage and investigating potential synergistic consequences of COMP and Aggrecan supplementation will potentially add to the translational value of our observations. This provides novel molecular clues for the optimization of IVB cartilage graft quality for cartilage repair in particular and for endochondral ossification-based cartilage regeneration techniques in general (Emans et al., 2011, 2012). Future work should be able to address the influence of IVB cartilage graft maturation on the pre-clinical outcome of cartilage repair.

## Data Availability Statement

All datasets presented in this study are included in the article/Supplementary Material.

## Ethics Statement

The animal study was reviewed and approved by Maastricht University animal ethical committee.

## Author Contributions

MC, MJ, DH, LR, PE, and TW: substantial contributions to the research design. MC, MJ, LP, AC, DS, and PE: substantial contributions to the acquisition of samples. MC, MJ, LP, AC, DS, PE, and TW: substantial contributions to analysis. MC, MJ, DH, LP, AC, DS, LR, PE, and TW: substantial contributions to the interpretation of the data, revising manuscript critically, and approval of the submitted and final versions. MC, MJ, PE, and TW: drafting the manuscript. All authors have read and approved the final submitted manuscript.

## Conflict of Interest

TW and MC are listed as inventor on patents: WO2017178251, WO2017178253, and US 20130123314. TW, PE, and LR have shares in Chondropeptix BV and are CSO, CMO, and CDO of Chondropeptix, respectively. DH is listed as inventor on US patent 9,133,259 and is President and CSO of Tesio Pharmaceuticals Inc.

The remaining authors declare that the research was conducted in the absence of any commercial or financial relationships that could be construed as a potential conflict of interest.
