# Supplementary material for: Aggrecan and COMP Improve Periosteal Chondrogenesis by Delaying Chondrocyte Hypertrophic Maturation
Source: Front Bioeng Biotechnol. 2020 Aug 28;8:1036. doi: 10.3389/fbioe.2020.01036 (PMC7483497; doi:10.3389/fbioe.2020.01036)
Supplement: Supplementary file 1 [file Image_1.pdf]

Supplementary information to accompany

"Aggrecan and COMP improve periosteal chondrogenesis by delaying chondrocyte hypertrophic maturation"

Marjolein M.J. Caron[1*]; Maarten P.F. Janssen[1*]; Laura Peeters[1]; Dominik R. Haudenschild[2]; Andy Cremers[1]; Don A.M. Surtel[1]; Lodewijk W. van Rhijn[1]; Pieter J. Emans[1*], Tim J.M. Welting[1*]

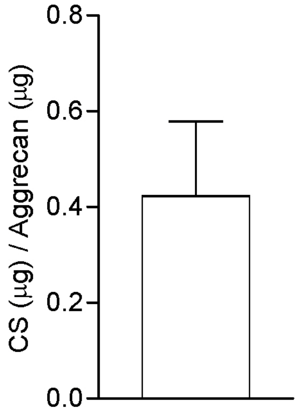

Supplementary Figure 1: Glycosaminoglycan (GAG) content of bovine Aggrecan from articular cartilage.

GAG content of bovine Aggrecan from articular cartilage used as supplement in this study was established by Dimethylmethylene Blue Assay (DMMB). The graph shows the chondroitin (CS) equivalent as a measure for GAG content per mass of Aggrecan.

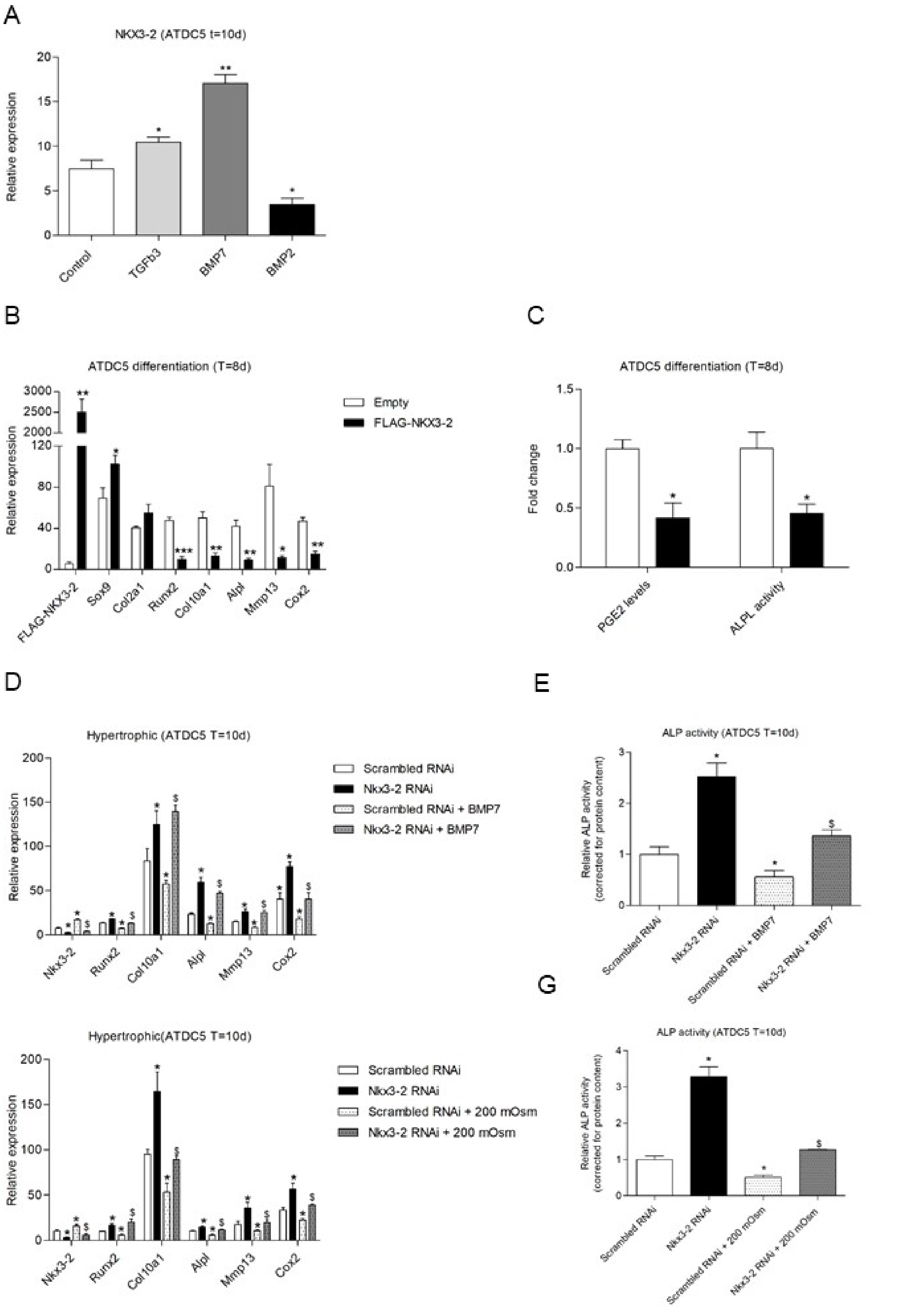

Supplementary Figure 2: Data supporting a role for NKX3-2 in hypertrophic differentiation during chondrogenic differentiation of ATDC5 cells.

These are data from experiments using the chondroprogenitor cell line ATDC5, which was differentiated in the chondrogenic lineage showing functional involvement of Nkx3-2 in hypertrophic differentiation in this model. The data show that expression of Nkx3-2 is increased at day 10 in ATDC5 differentiation by the growth factors TGFβ3 and BMP7, but decreased by BMP2 (Figure 2A). Overexpression of FLAG-Nkx3-2 by polyethyleneimine-mediated transfection of an Nkx3-2 p3XFLAG-CMV-7.1 expression vector (1000 ng of plasmid/well) resulted in decreased expression of chondrocyte hypertrophic markers and decreased PGE_2 levels in the culture supernatant, as well as reduced ALP enzyme activity (Figure 2B and 2C). Reducing Nkx3-2 levels by RNAi (100 nM siRNA; transient transfection on day 0 and 4 in ATDC5 differentiation) resulted in increased expression of hypertrophic markers Runx2, Col10a1, Alpl, Mmp13 and Cox-2, as well as functional ALP enzyme activity (Figures 2D-G; white vs. black bars). Addition of BMP7 (Figures 2 D/E; white dotted vs. grey dotted) or increasing the osmolarity of the culture medium (Figure2 F/G; white dotted vs. grey dotted) with 200 mOsm (using NaCl) during chondrogenic differentiation reduced hypertrophic differentiation of ATDC5 cells in an Nkx3-2 dependent manner. Considering the conserved and central "switch" function of Nkx3-2 during the hypertrophic phase of chondrogenic differentiation (1-4), and combined with the above ATDC5 data, we speculate that a similar Nkx3-2-dependent mechanism might be active in rabbit periosteal chondrogenesis and potentially providing an underlying mechanism behind our observed COMP and Aggrecan-dependent modulation of chondrocyte hypertrophic differentiation.

References

1. M. M. Caron, P. J. Emans, A. Cremers, D. A. Surtel, M. M. Coolsen, L. W. van Rhijn and T. J. Welting: Hypertrophic differentiation during chondrogenic differentiation of progenitor cells is stimulated by BMP-2 but suppressed by BMP-7. *Osteoarthritis Cartilage*, 21(4), 604-13 (2013) doi:10.1016/j.joca.2013.01.009
2. M. M. Caron, P. J. Emans, D. A. Surtel, P. M. van der Kraan, L. W. van Rhijn and T. J. Welting: BAPX-1/NKX-3.2 acts as a chondrocyte hypertrophy molecular switch in osteoarthritis. *Arthritis Rheumatol*, 67(11), 2944-56 (2015) doi:10.1002/art.39293
3. S. Provot, H. Kempf, L. C. Murtaugh, U. I. Chung, D. W. Kim, J. Chyung, H. M. Kronenberg and A. B. Lassar: Nkx3.2/Bapx1 acts as a negative regulator of chondrocyte maturation. *Development*, 133(4), 651-62 (2006) doi:10.1242/dev.02258
4. S. Yamashita, M. Andoh, H. Ueno-Kudoh, T. Sato, S. Miyaki and H. Asahara: Sox9 directly promotes Bapx1 gene expression to repress Runx2 in chondrocytes. *Exp Cell Res*, 315(13), 2231-40 (2009) doi:10.1016/j.yexcr.2009.03.008
